# Hospitalized Patients Accessing Information on Prescribed Medications from the Bedside Terminal: A Cross-Sectional Study

**DOI:** 10.3390/ijerph17134850

**Published:** 2020-07-06

**Authors:** Jungwon Cho, Seungyeon Kim, Sangyoon Shin, Hyejin Yoo, Gi Hyue Park, Eunha Jeon, Eunsook Lee, Ho-Young Lee, Euni Lee

**Affiliations:** 1 College of Pharmacy & Research Institute of Pharmaceutical Sciences, Seoul National University, Gwanak-ro 1, Gwanak-gu, Seoul 08826, Korea; xcully@snu.ac.kr (J.C.); gsy92@snu.ac.kr (S.K.); snine9@snu.ac.kr (S.S.); hyejin0414@snu.ac.kr (H.Y.); gihyuepark@snu.ac.kr (G.H.P.); 2 Department of Pharmacy, Seoul National University Bundang Hospital, 82, Gumi-ro 173, Bundang-gu, Seongnam-si, Gyeonggi-do 13620, Korea; escduck@snubh.org; 3 Office of Digital Medicine, Seoul National University Bundang Hospital, 82, Gumi-ro 173, Bundang-gu, Seongnam-si, Gyeonggi-do 13620, Korea; siszoo@snubh.org; 4 Department of Nuclear Medicine, Seoul National University Bundang Hospital, Seoul National University College of Medicine, 82, Gumi-ro 173, Bundang-gu, Seongnam-si, Gyeonggi-do 13620, Korea

**Keywords:** health care information technology, patient portals, personal health record, patient empowerment

## Abstract

Studies have documented the impact of various types of health care information technology (HIT) on patient outcomes. However, literature on the HIT products is largely for outpatients and little is known about those for hospitalized patients. In 2014, a Korean hospital developed an inpatient portal known as the Smart Bedside Station (SBS). A retrospective cross-sectional study was conducted to evaluate the associated factors for accessing the medication view menu (*Today’s Medication*) on the SBS using data from October 2018 through September 2019. A root cause analysis with expert review was conducted to identify additional barriers for accessing the medication view menu. Approximately 92.58% of the study population accessed the SBS at least once during their hospital stay. However, 99.20% of accessed patients used the SBS for entertainment purposes (e.g., television) and 40.16% viewed the medication information. Younger age, higher education, and certain jobs were significant associated factors for accessing the medication information. In conclusion, this study revealed strong associations between accessing the medication view menu on the SBS and a number of associated factors. Based on the results, further research is warranted to suggest new items to access the medication view menu by hospitalized patients.

## 1. Introduction

In recent decades, health care information technology (HIT) has evolved consistently to focus on improving the quality, safety, and efficiency of health care delivery [1]. In the era of the Fourth Industrial Revolution, many HIT devices have been introduced and highly intelligent technology utilizing artificial intelligence or innovative treatments will change the health care environment [2,3,4,5]. Although many HIT initiatives were initially developed from the perspectives of health care providers and administrators, more recent development has focused on incorporating the perspectives of patients as the end users [6]. Despite this, the literature describing how these advancements in HIT products impacted the lives of the end users remains limited. The field of HIT emerged with the synergistic development of electronic health records; personal health records and other associated technology such as telemedicine and telehealth [7]. Within the last 25 years, new medical technologies, including patient portals, have emerged for delivering patient-centered care. Consequently, the roles of patients have changed from passive to autonomous care recipients and competent partners who actively engage in their own treatment [8,9].

Patient-centered care is the practice of caring for patients (and their caregivers) in ways that are meaningful and valuable to them. The definition of patient-centered care provided by the Institute of Medicine [10] and a guiding principle described by the Picker Institute [11] suggests that health care institutions should provide information, communication, and education to patients about their clinical status, progress, prognosis, and processes of care, taking into consideration their individual preferences, needs, and values. While various types of HITs are available for increasing medication adherence [12,13,14], the majority of them have focused on outpatient management, and very little is known regarding their use in inpatient care settings [15,16]. For inpatient care, access to clear instructions is essential for medication adherence, which in turn affects clinical outcomes [17,18,19].

**Figure 1:**
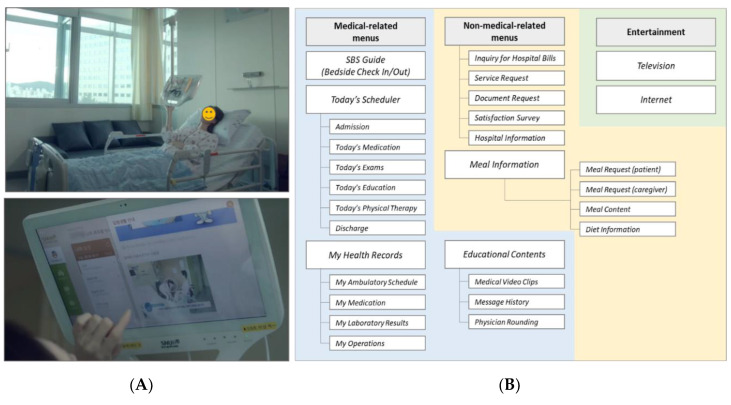
(**A**) Screenshots of using the Smart Bedside Station (SBS) (available from the website of the Seoul National University Bundang Hospital, http://www.snubh.org) and (**B**) key menus on the SBS (medical-related, non-medical-related and entertainment).

Patient portals can be considered as effective tools for patients by allowing them to acquire information on clinical conditions and medications, and actively participate in their own treatment process [14,15,20]. Recently, bedside hardware known as the Smart Bedside Station (SBS) has been developed using comprehensive user research at a Korean hospital [21]. The SBS, which is a bedside-attached tablet (Figure 1A), allows a patient to choose from a menu including options on entertainment, medical-related information, and non-medical-related information in a similar way to the patient portals. An exploratory study on the SBS focused on its entertainment components [22,23], but detailed information is lacking on the accessibility of medication information (i.e., *Today’s Medication*) within the SBS by the patients. Therefore, we have conducted a retrospective cross-sectional study with the primary objective of evaluating the associated factors for accessing *Today’s Medication* after describing the usage patterns of the SBS by the hospitalized patients. We also performed a root cause analysis with the secondary research objective to identify additional barriers for accessing the *Today’s Medication* menu that could not be evaluable from the descriptive analysis of the SBS usage patterns.

## 2. Materials and Methods

A detailed description about the SBS, the study environment, pictures of the SBS and the menu components, step-by-step descriptions on our research process including a retrospective cross-sectional evaluation for accessing *Today’s Medication* on the SBS, and the subsequent analysis, a root cause analysis using a fishbone diagram on potential causes for the low numbers accessing *Today’s Medication* were provided as below.

### 2.1. Study Site and the Smart Bedside Station (SBS)

Seoul National University Bundang Hospital (SNUBH) is a 1350-bed academic teaching hospital that offers comprehensive care at 33 ambulatory clinics serving approximately 1.6 million outpatients annually. Since 2010, SNUBH features fully digitized HIT and the Health Care Information and Management System Society certified it as having reached Stage 7, the highest stage of the certification offered by the society located in the United States [24]. SNUBH utilized its advanced HIT for inpatient care to develop the SBS in 2014, and details on the SBS are available elsewhere [21,22].

The SBS, initially developed reflecting user experience, consisted of three menu categories, namely medical-related menus, non-medical-related menus, and entertainment (Figure 1B). *Bedside Check In & Out* provided overall guidance for hospital life and amenities. *Today’s Scheduler* detailed a patient’s daily schedule related to treatment, such as imaging studies, lab tests, and physical therapy. One submenu of *Today’s Scheduler* was *Today’s Medication* that provided detailed information about each patient’s prescribed medications daily and included pill images, a dosage summary, usage notes, and a comprehensive description of adverse effects. To start the SBS, the patients were requested to log in with the patient’s identification number or scan the radio-frequency identification band.

### 2.2. Study Process

Our study aimed to describe the usage patterns of SBS and to evaluate the associated factors for accessing the *Today’s Medication* menu by analyzing the SBS access data. Based on the results from the analysis, we conducted a root cause analysis using a fishbone diagram to identify additional barriers for accessing *Today’s Medication* that could not be evaluable from the descriptive analyses of the SBS usage patterns.

#### 2.2.1. Analysis of the SBS Access Data

The SBS access data were compiled for patients who were hospitalized during the one-year period from 1 October 2018 to 30 September 2019. The study population excluded hospitalized patients who were in special units such as intensive care units, emergency rooms, and delivery wards, or those discharged within a day. The SBS usage profiles collected included demographic characteristics and the number of page calls per SBS menu made by each patient during their hospital stay. The SBS access data were tabulated by accessing *Today’s Medication* menu and the potential associated factors for accessing the menu were evaluated by comparing patients’ demographic and admission characteristics.

#### 2.2.2. Root Cause Analysis

We set the low numbers accessing the medication view as an event, and evaluation was performed based on the analysis results of the SBS access data. A total of seven pharmacists, each having over 10 years of experience as hospital pharmacists, were invited to review the findings related to the SBS usage patterns. We identified a list for the low numbers accessing *Today’s Medication* using a fishbone diagram and subsequently devised potential solutions in accessibility, patient engagement, and enhancing care quality [17].

### 2.3. Data Analysis

The analysis of the study focused on estimating the frequency of patients accessing the *Today’s Medication* menu in the SBS and evaluating its associated factors. Descriptive statistics were used to summarize patients’ demographic characteristics overall. The characteristics between the two groups of patients who did and did not access *Today’s Medication* menu were compared using a Pearson’s chi-squared test and the student’s t-test, respectively, for categorical and continuous variables. Multivariable logistic regression analysis was constructed to analyze associated factors for accessing *Today’s Medication* menu (yes/no), set as the dependent variable. The adjusted ORs with 95% confidence intervals (CIs) were determined while adjusting confounders such as gender, age, education level, and job type. All statistical analyses were performed using SAS Version 9.4 (SAS Institute Inc., Cary, N.C., USA), and the level of statistical significance was set at 95% (*p* < 0.05).

### 2.4. Ethical Board Approval

This retrospective study was approved by the institutional review board of SNUBH (IRB number: B-1907/552-102), and a waiver for written consent was obtained from the board.

## 3. Results

We presented our findings on the SBS usage patterns and associated factors in tables and provided a fishbone diagram to demonstrate all potential barriers for the low numbers accessing *Today’s Medication* on the SBS.

### 3.1. SBS Usage Patterns

During the 12-month study period, from a total of 36,931 patients, 34,189 (92.58%) used the SBS at least once during their hospital stay. Table 1 represents the demographic characteristics of the study population. Among the patients who accessed the SBS, patients who experienced entertainment (e.g., television) were 33,914 (99.20%). However, patients who accessed medical-related menus were 22,591 (66.08%), of which 13,732 (40.16%) accessed *Today’s Medication*.

Table 2 shows patient characteristics by their access status to *Today’s Medication* menu during their hospital stay. Patients who accessed *Today’s Medication* during their hospital stay were significantly younger than the patients who did not (50.79 ± 21.04 years vs. 59.65 ± 20.43 years, *p* < 0.05). In fact, nearly 72% of patients over the age of 65 did not access *Today’s Medication*. However, with respect to the department of hospitalization, the only department that had a higher proportion of patients who accessed the service compared to patients who did not was Obstetrics and Gynecology. Regarding education level, the group with college education or above were the highest proportion of the patients who accessed the service (6004, 48.49%). Regarding job type, patients who were company workers, students, and in military service accessed the service by over 50% among each group. On the other hand, the rate of access was the lowest for patients without a job (31.00%), followed by housewives (37.64%).

### 3.2. Associated Factors for Accessing Today’s Medication

Several demographic characteristics were found to be associated with patients’ use of *Today’s Medication* at least once during their hospital stay (Table 3). Patients who were aged 65 or younger had an increased probability of accessing *Today’s Medication*. Patients who were aged 20–39 had significantly increased probability of accessing *Today’s Medication* compared to patients who were aged over 65 (OR = 2.631, 95% CI 2.409–2.874). With respect to education level, patients who had graduated at college or above (OR = 1.543; 95% CI 1.419–1.677) or had middle to high school education (OR = 1.153; 95% CI 1.067–1.247) had an increased probability of accessing *Today’s Medication*, compared to patients with lower education. Regarding job type, patients who were professionals (OR = 1.247; 95% CI 1.125–1.382), company workers (OR = 1.418; 95% CI 1.303–1.544), students (OR = 1.617; 95% CI 1.324–1.974) and in military service (OR = 1.724; 95% CI 1.249–2.379) were more likely to access *Today’s Medication* compared to patients without a job.

### 3.3. Root Cause Analysis Using a Fishbone Diagram

**Figure 2:**
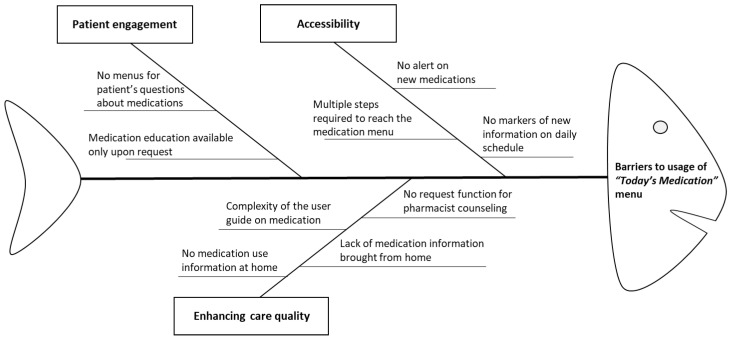
Fishbone diagram outlining barriers to usage of *Today’s Medication.*

Starting with the low numbers accessing *Today’s Medication*, a fishbone diagram (Figure 2) was created to outline the potential barriers to accessing *Today’s Medication*. Subsequently devised potential solutions displayed in accessibility, patient engagement, and enhancing care quality.

## 4. Discussion

This study analyzed patient usage of the SBS, a bedside patient portal, with the goal of identifying factors that were associated with the usage of the medication view menu on the SBS. Our study had several strengths. First, we used real-world data from inpatients to analyze the SBS usage patterns over a one-year period and showed the results in a simple and intuitive manner. Then, we conducted a root cause analysis to identify additional barriers for accessing the *Today’s Medication* menu that could not be evaluable from the descriptive analysis of the SBS usage patterns in a systematic way.

Our study showed that the most frequently used menu was predominantly on entertainment and only 40.16% patients visited the medication information view among all patients who used the SBS in their hospital stay. Based on the results, the number of patients who accessed *Today’s Medication* was relatively low compared to the number who accessed the entertainment menu on the SBS and the multivariable logistic regression analysis showed that older age, low educational level, and certain jobs were significant associated factors. Although our study only evaluated accessing the *Today’s Medication* without assessing patients’ actual ability to seek, find, or understand medication information, findings from our study related to the low numbers accessing *Today’s Medication* and older age or low education were consistent with previous studies. As the ability to use technology was considered as one of multiple skills for an individual to be health literate [25], linking low health literacy and the adoption of health behaviors [26,27], poor access to electronic health resources [25,28], and health literacy problems among older adults, particularly those who have limited education [29], we believe that the recorded access numbers of *Today’s Medication* from our study can be considered as one of the capacity indicators for using technology.

We only measured SBS usage by patients accessing each menu at least once during their hospital stay, as the primary focus of our study was to estimate patient access rates for the medication information. However, persistence in patient utilization can be evaluated as an additional measure by counting the number of times they issued a page call for each menu in the future.

We believe a HIT such as the SBS could serve as a potentially powerful tool to aid the transition of care, which can provide patients with sufficient knowledge about their medications during their hospital stay and their discharge medications. As the World Health Organization defined empowerment as “a process through which people gain greater control over decisions and actions affecting their health” [30], a HIT product operated by the patients, such as the SBS from our study, with easy access to information can be an important tool to empower patients. Although previous trials mainly evaluated web-based platforms used with tablet computers [14] and many studies on patient portals have focused on outpatient management [15,16], findings from our study added important insights, as our study evaluated whether patients accessed and utilized a HIT product as a bedside hardware and the focus of our evaluation was based on the hospitalized patients to enhance patient empowerment. In addition, we believe that our study can serve as a research basis for future studies for medication tracking and shared decision making [31], and can improve clinical outcomes [20], through patient empowerment by enhancing access to medication information.

Based on the findings from our study, the following recommendations can be made to enhance the accessibility of *Today’s Medication* on the SBS in three domains as suggested in the literature [14]—accessibility, patient engagement, and enhancing care quality for inpatient portal use. In terms of accessibility, we focused on directly guiding the patients to the *Today’s Medication* menu from the main menu or other menus such as television on the SBS. In terms of accessibility, we make two suggestions—insert an alert marker signifying that information has been added on *Today’s Scheduler* and add real-time pop-ups when new medications are added under *Today’s Medication*—to guide the patients while they are viewing other menus such as entertainment. Regarding patient engagement, we suggest voluntary enrollment for medication education programs and offering writing notepads to patient groups with increased probability of accessing *Today’s Medication* (younger age, higher education level, and certain jobs). In contrast, for the patients who had a lower probability of accessing *Today’s Medication*, new items for enhancing care quality were derived—providing detailed medication use information, medication counseling by a designated pharmacist and sending a URL of medication use information via text message were suggested.

### Limitations

This study also has limitations. First, our study using a cross-sectional study design with inherent limitations such as the non-randomized, uncontrolled study design could only explain associations between accessing the *Today’s Medication* menu and other covariates. The second limitation of our study was related to the data source. Our study used electronic medical records and SBS access data with limited data elements. As a secondary data analysis, this study was limited in understanding of the multitude of explanatory factors. To overcome the limitation, we conducted a root cause analysis using a fishbone diagram with the experts. Thirdly, our study did not evaluate interrater reliability for the root cause analysis. Therefore, further study may be needed to address detailed causes of the access issues and propose comprehensive solutions. Lastly, our study could not differentiate the SBS operator. The SBS device can be started with a log in using the patient’s identification code or by scanning a radio-frequency identification band. In reality, both patients or their caregivers can start the device. Although our study could not determine who the actual beneficiaries of the medication knowledge were, we believe that our study could provide insights on the accessibility of the medication information via a HIT product during the hospitalization and serve as a basis for the upcoming era of the advanced medical and health technology.

## 5. Conclusions

The SBS performs various medical-related, non-medical-related, and entertainment functions. Based on the analysis of inpatient data, however, we found that many patients utilized the entertainment functions far more frequently than *Today’s Medication* on the SBS. Additionally, *Today’s Medication* and certain characteristics such as age, education level, and job type were strongly associated factors for accessing *Today’s Medication.* We believe that this retrospective cross-sectional study could serve as a stepping stone for further studies about patient portals and improve the HIT.

## Figures and Tables

**Table 1:** Characteristics of the study population (N = 36,931)**.**

Characteristics	Mean (SD)	N (%)
Age, mean ± SD	55.78 ± 21.35	
Under 20		2966 (8.03)
20–39		4810 (13.02)
40–64		14,444 (39.11)
Over 65		14,711 (39.83)
Gender (men)		18,492 (50.07)
Length of stay, mean ± SD	11.76 ± 17.82	
Department of hospitalization		
Surgery		13,075 (35.40)
Internal medicine		12,991 (35.18)
Obstetrics and gynecology		2889 (7.82)
Urology		1933 (5.23)
Pediatric		1656 (4.48)
Others		4387 (11.88)
Education level *		
Less than elementary school		5184 (16.07)
Middle to high school education		13,571 (42.07)
College education or above		13,503 (41.86)
Job type *		
No job		9254 (28.69)
Professionals		2451 (7.60)
Business owners		3509 (10.88)
Company workers		4890 (15.16)
Housewives		8126 (25.19)
Students		1411 (4.37)
Military service		200 (0.62)
Others ^a^		2416 (7.49)

Data are presented as the mean ± SD with range (minimum, maximum) for age and length of stay, and n (%) for other variables; SD: standard deviation. * There were 4673 missing values for education level and 4674 missing values for job type. ^a^ Others included farmer, government employee, construction worker, commercial driver, clergy, freelancer, etc., in rank order.

**Table 2:** Patient characteristics by whether they accessed the *Today’s Medication* menu (N = 34,189)**.**

Characteristics	Total SBS Use (n = 34,189)	N (%)	
		Accessed ^a^ (n = 13,732)	Did Not Access (n = 20,457)
Age, mean ± SD		50.79 ± 21.04	59.65 ± 20.43
Under 20	2668	1290 (48.35)	1378 (51.65)
20–39	4268	2547 (59.68)	1721 (40.32)
40–64	13,491	6007 (44.53)	7484 (55.47)
Over 65	13,762	3888 (28.25)	9874 (71.75)
Gender (men)	17,230	6784 (49.40)	10,446 (51.06)
Department of hospitalization			
Surgery	12,410	4999 (40.28)	7411 (59.72)
Internal medicine	12,146	4276 (35.21)	7870 (64.79)
Obstetrics and gynecology	2672	1427 (53.41)	1245 (46.59)
Urology	1884	831 (44.11)	1053 (55.89)
Pediatric	1512	735 (48.61)	777 (51.39)
Others	3565	1464 (41.07)	2101 (58.93)
Education level *			
Less than elementary school	4827	1445 (29.94)	3382 (70.06)
Middle to high school education	12,651	4600 (36.36)	8051 (63.64)
College education or above	12,383	6004 (48.49)	6379 (51.51)
Job type *			
No job	8614	2670 (31.00)	5944 (69.00)
Professionals	2275	1137 (49.98)	1138 (50.02)
Business owners	3292	1322 (40.16)	1970 (59.84)
Company workers	4576	2460 (53.76)	2116 (46.24)
Housewives	7497	2822 (37.64)	4675 (62.36)
Students	1230	662 (53.82)	568 (46.18)
Military service	166	98 (59.04)	68 (40.96)
Others ^b^	2210	875 (39.59)	1335 (60.41)

Data are presented as the mean ± SD with range (minimum, maximum) for age and n (%) for other variables; SD: standard deviation. * There were 4328 missing values for education level and 4329 missing values for job type. ^a^ Patients who accessed *Today’s Medication* menu during their hospital stay. ^b^ Others included farmer, government employee, construction worker, commercial driver, clergy, freelancer, etc., in rank order.

**Table 3:** Associated factors for patient accessing the *Today’s Medication* menu (N = 29,856).

Characteristics	Adjusted OR	95% CI
Gender		
Women	1.00 (reference)	
Men	0.983	0.925–1.044
Age		
Over 65	1.00 (reference)	
Under 20	1.856 *	1.478–2.331
20–39	2.631 *	2.409–2.874
40–64	1.714 *	1.612–1.823
Education level		
Less than elementary school	1.00 (reference)	
Above graduate education	1.543 *	1.419–1.677
Middle to high school education	1.153 *	1.067–1.247
Job type		
No job	1.00 (reference)	
Professionals	1.247 *	1.125–1.382
Business owners	1.088	0.995–1.190
Company workers	1.418 *	1.303–1.544
Housewives	1.055	0.974–1.143
Students	1.617 *	1.324–1.974
Military service	1.724 *	1.249–2.379
Others ^a^	1.103	0.996–1.221

* *p* < 0.05; OR: odds ratio; CI: confidence interval. ^a^ Others included farmer, government employee, construction worker, commercial driver, clergy, freelancer, etc., in rank order.
